# Supplementary material for: Molecular Characterization of a Transcriptional Regulator GntR for Gluconate Metabolism in Industrial 2-Ketogluconate Producer Pseudomonas plecoglossicida JUIM01
Source: Microorganisms. 2025 Jun 15;13(6):1395. doi: 10.3390/microorganisms13061395 (PMC12195362; doi:10.3390/microorganisms13061395)
Supplement: Supplementary file 1 [file microorganisms-13-01395-s001.zip › microorganisms-3606271-supplementary.pdf]

Supplementary materials

Molecular Characterization of a Transcriptional Regulator GntR for Gluconate Metabolism in Industrial 2-Ketogluconate Producer *Pseudomonas plecoglossicida* JUIM01

Table S1. Primers used in this study

Primers	Sequences (5′→3′)	Description (restriction sites)
GntR-F	GGAATTCCATATGATGTCCCGCACAGGCTCGC	To amplify the *gntR* gene (*Nde* I)
GntR-R	CCGCTCGAGTCAGGTGCTTTCGCGAACCATC	To amplify the *gntR* gene (*Xho* I)
T7	TAATACGACTCACTATAGG	Universal primers for vector pET-28a (+) sequencing
T7 Ter	GCTAGTTATTGCTCAGCGG	
Δ*gntR*-K1	CCGGAATTCCATGGTTGAAGTGCCTCGATCTT	To amplify the upstream homologous fragment of the *gntR* gene (*Eco*R I)
Δ*gntR*-K2	AGGTCCTGCTGCTCGGTCCGGGGCGACGGTACTGAT	
Δ*gntR*-K3	ATCAGTACCGTCGCCCCGGACCGAGCAGCAGGACCT	To amplify the downstream homologous fragment of the *gntR* gene (*Hin*d III)
Δ*gntR*-K4	CCCAAGCTTCTTCGATGTGGTGTTGACCGACT	
16s-F	TCGGTACAGAGGGTTGCCAAG	The following primers are used for RT-qPCR
16s-R	GTGTACAAGGCCCGGGAACG	
*oprB*_1-F	ATCATCGCATACGCATCAGG	
*oprB*_1-R	CGCCGACATAGTCAAGGGTG	
gltK-F	ACAACGATCCGGCCAACC	
gltK-R	TCCCATTCGCCCTCCACT	
gltG-F	GCTTCAAGACACCCGAGGAT	
gltG-R	GCAACCGAACAGCAACAGG	
gltF-F	AACGCTGGCTCCCGAAAC	
gltF-R	GCTGGAGTGGGTGAAGGACA	
gltB-F	AGATCATGGGCGACTGGG	
gltB-R	CCTTCTGCGACTGCTGGGT	
gtrS-F	GCGGGCGTTCAACAGCAT	
gtrS-R	CGGCGAGGATCTCAAGCA	
gltR-F	CCACCCGCAGCAAATCCTC	
gltR-R	TCAGCAGGCCGGGCAGACAT	
ptxS-F	CCCTTGGTCGGTGGCGGTAT	
ptxS-R	TGGAGCCTCGCACGATGAGC	
gntK-F	GGCGATGCCTTTCACCCT	
gntK-R	CAGGAATACGAAACCAAGACCC	
gntP-F	ATCGTGCGCCAGTGGATG	
gntP-R	GCCAGGAGGATCGGTGAGAT	
kguD-F	GCTCACCGAAACCACTGCC	
kguD-R	ACGATGCCCAGCGTCTTGC	
kguT-F	TGGCTGAAGGCACAGGAGA	
kguT-R	TGGCAACCAGAGCACGAA	
kguK-F	AGCGACCCGCAAGTGGAA	
kguK-R	CCGCCAGGGCATTGATTT	
kguE-F	TCAGCCTGTCGAGCTTCGG	
kguE-R	GGGAAACCTTCAATGCCACC	

Table S2. The promoter region sequences of the genes *gntR*, *gntK*, and *gntP*

Genes	The promoter region sequences (5′→3′)
gntR	TGGGGCGACCGGTGGTACGCGAGCCTGTGCGGGACATGGGAGTTGTAATTTTACTA CTTGCCAGAAAGGGGAACGGCCACTAAGGTAGCGCTGTCTCAGGACACAGGCAACG TAAAACTTTAGCGGCTATGTGCCCAGCGTCCATACTGCG (151 bp)
gntK	TGCCCAGCGTCCATACTGCGTGAAGACAAGACCAATAACACCCGGGAGGGTGGCGC TTGCATCCTCGTTTCGTCGCCAGCGAGACAGCGCTATCTTACCCCGCAGGAGGTACT GATGAACTCTCCCCTGTCTGCAATAGTGGTGATGGGTGTG (153 bp)
gntP	TGCCGGCGGTTTGTCCGGCTCACCAAAGACAGCGCTGTCTCGACACCTGAGCGTGTC GAACGTTTCGCCAAAACAACGATAAGATCGAGGCACTTCAACCATGTTCGGACTGG CTACTGATACCTTCCTGCTGCTCGACGCCCTGGTGACCATCGTCGGGC (161 bp)

Table S3. Secondary structure analysis of the recombinant PpGntR

Wavelength	190-260 nm	195-260 nm	200-260 nm	205-260 nm	210-260 nm
α-Helix	33.8%	32.0%	30.1%	32.2%	31.2%
Antiparallel	4.7%	12.9%	13.0%	13.1%	13.0%
Parallel	8.8%	6.9%	5.7%	5.8%	5.7%
β-Turn	16.3%	16.2%	16.2%	17.1%	17.5%
Random coil	33.1%	30.1%	30.3%	31.6%	31.3%
Total Sum	96.7%	98.1%	95.4%	99.9%	98.8%

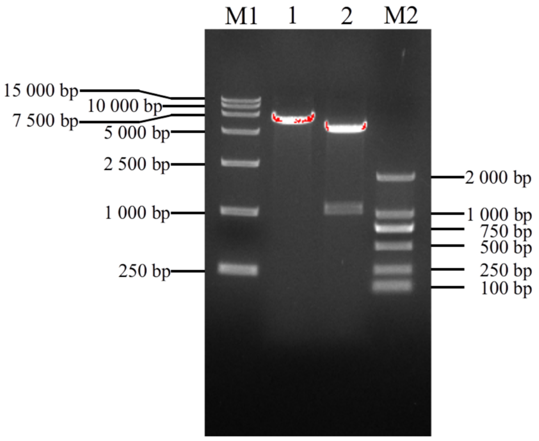

Figure S1. The recombinant plasmid pET-28a(+)-*gntR* was verified through the digestion of the gene fragment with *Nde* I and *Xho* I. Lane M1, 15000 bp molecular weight marker; Lane 1, the single enzyme (*Xho* I) digestion product of the recombinant plasmid pET-28a(+)-*gntR*; Lane 2, the double enzyme (*Nde* I/*Xho* I) digestion products of the recombinant plasmid pET-28a(+)-*gntR*; Lane M2, 2000 bp molecular weight marker.

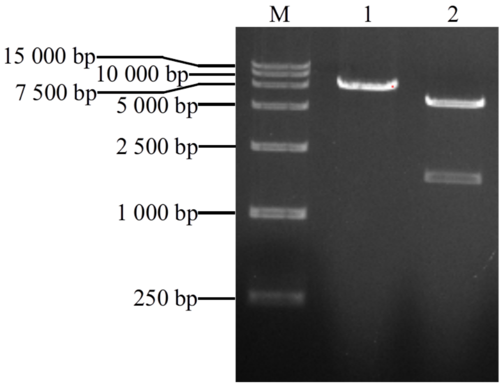

Figure S2. The recombinant plasmid pK18*mobsacB*-Δ*gntR* was verified through the digestion of the gene fragment with *Eco*R I and *Hin*d III. Lane M1, 15000 bp molecular weight marker; Lane 1, the single enzyme (*Eco*R I) digestion product of the recombinant plasmid pK18*mobsacB*-Δ*gntR*; Lane 2, the double enzyme (*Eco*R I/*Hin*d III) digestion products of the recombinant plasmid pK18*mobsacB*-Δ*gntR*.

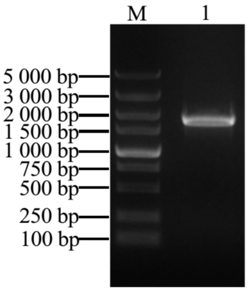

Figure S3. The colony PCR verification of the gene-deletion strain. Lane M, 5000 bp molecular weight marker; Lane 1, the incomplete *gntR* gene fragment (Δ*gntR*).

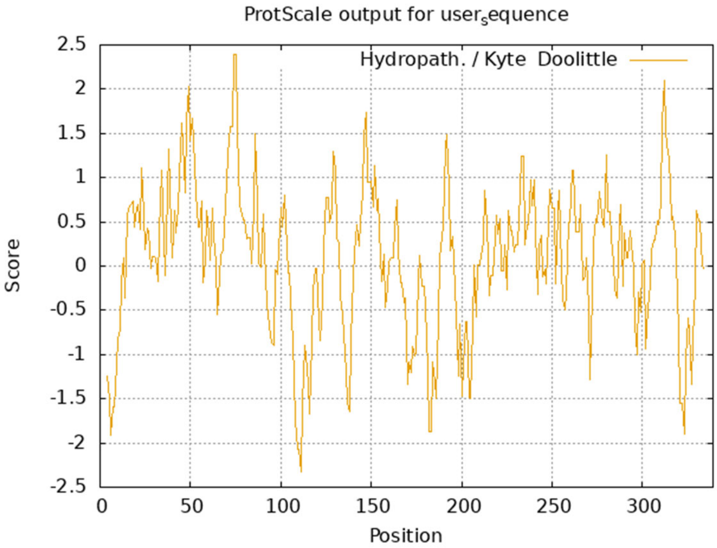

Figure S4. The hydropathy plot of the GntR of *Pseudomonas plecoglossicida* JUIM01. The result shows a grand average of hydropathicity (GRAVY) of 0.071.

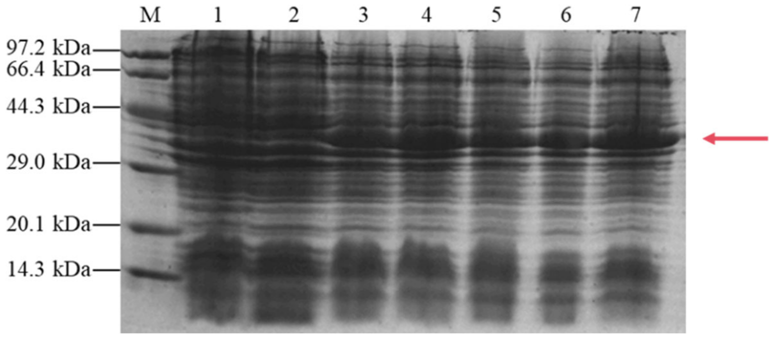

Figure S5. The effect of IPTG concentration on the expression of GntR in *E. coli* BL21(DE3)/pET-28a(+)-*gntR* lysate supernatants. Lane M, protein marker; Lane 1, *E. coli* BL21(DE3)/pET-28a(+) (0 mM IPTG); Lane 2, *E. coli* BL21(DE3)/pET-28a(+)-*gntR* (0 mM IPTG); Lane 3-7, *E. coli* BL21(DE3)/pET-28a(+)-*gntR* (IPTG concentration of 0.2, 0.4, 0.6, 0.8 and 1 mM).

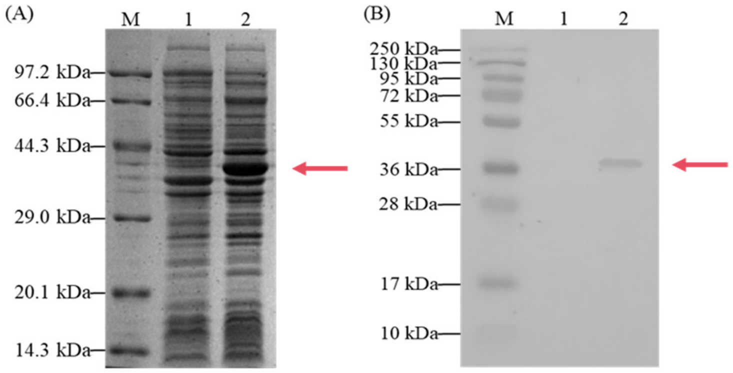

Figure S6. SDS-PAGE (**A**) and Western blot (**B**) analyses of the whole cell proteins of *E. coli* BL21(DE3)/pET-28a(+)-*gntR*. Lane M, protein marker; Lane 1, *E. coli* BL21(DE3)/pET-28a(+); Lane 2, *E. coli* BL21(DE3)/pET-28a(+)-*gntR*.

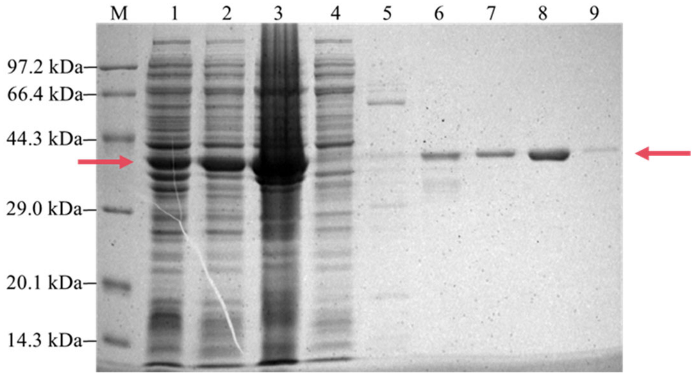

Figure S7. Purification of the recombinant PpGntR. Lane M, protein marker; Lane 1, cell lysate; Lane 2, supernatant of cell lysate; Lane 3, debris of cell lysate; Lane 4, penetrating fluid after up-

sampling (supernatant of cell breakage solution); Lane 5-9, eluent with buffer containing 100, 150, 200, 250, and 500 mM of imidazole.

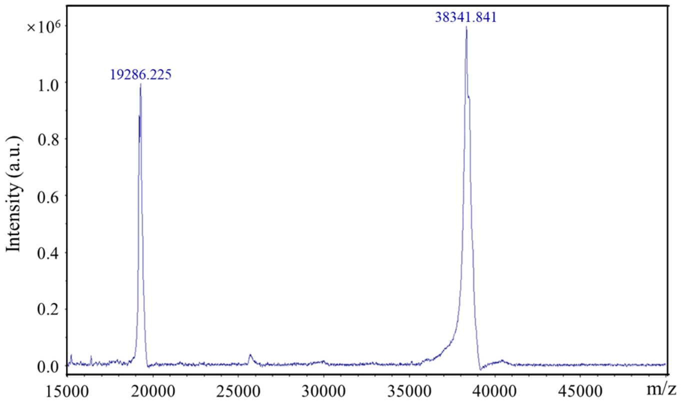

Figure S8. Determination of molecular weight of the recombinant PpGntR by MALDI-TOF-MS.

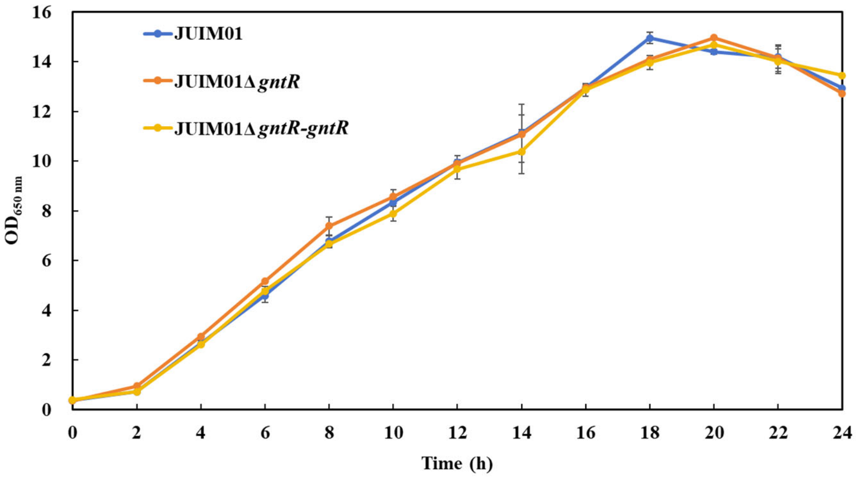

Figure S9. Comparison of the cell growth of *Pseudomonas plecoglossicida* JUIM01, JUIM01Δ*gntR*, and JUIM01Δ*gntR-gntR*.
